# Outdoor air pollution and psychiatric symptoms in adolescents: a study of peripheral inflammatory marker associations

**DOI:** 10.3389/fpsyt.2025.1588964

**Published:** 2025-05-23

**Authors:** Clara G. Zundel, MacKenna M. Shampine, Alexander Jakubiec, Christine Lewis, Cole Brokamp, Jeffrey R. Strawn, Tanja Jovanovic, Patrick H. Ryan, Eric Woodcock, Zhao Yang, Michael Petriello, Hilary Marusak

**Affiliations:** ^1^ Department of Psychiatry and Behavioral Neurosciences, Wayne State University School of Medicine, Detroit, MI, United States; ^2^ Department of Pediatrics, University of Cincinnati College of Medicine, Cincinnati, OH, United States; ^3^ Division of Biostatistics and Epidemiology, Cincinnati Children’s Hospital Medical Center, Cincinnati, OH, United States; ^4^ Anxiety Disorders Research Program, Department of Psychiatry and Behavioral Neuroscience, University of Cincinnati, Cincinnati, OH, United States; ^5^ Merrill Palmer Skillman Institute for Child and Family Development, Wayne State University, Detroit, MI, United States; ^6^ Institute of Environmental Health Sciences, Wayne State University, Detroit, MI, United States; ^7^ Department of Pharmacology, Wayne State University School of Medicine, Detroit, MI, United States

**Keywords:** outdoor, air pollution, particulate matter, adolescence, anxiety, inflammation

## Abstract

**Introduction:**

Fine particulate matter (PM_2.5_) air pollution is associated with increased internalizing symptoms (e.g. depressive and anxiety symptoms), particularly during adolescence—a critical period for the emergence of anxiety disorders and vulnerability to neurotoxicants. Preclinical studies suggest that inflammation, including cytokines, reactive proteins, and lipid mediators, may explain the link between PM_2.5_ and psychiatric risk. However, growing evidence suggests that these relationships may differ by sex, with females potentially more vulnerable to the effects of air pollution on psychiatric symptoms, though the underlying mechanisms remain unclear.

**Methods:**

This study examined the relationships among recent (past-month) PM2.5 exposure, peripheral inflammatory markers, and anxiety and depressive symptoms in 78 adolescents (M ± SD = 13.3 ± 2.3 years, 48.7% female) from the Detroit, MI area.

**Results:**

Higher PM_2.5_ concentrations were significantly associated with elevated levels of inflammatory lipid mediators: PGE2, 12(S)-HETE, 12(S)-HEPE, and 15(S)-HETE. A significant PM_2.5_-by-sex interaction was observed for IL-6, with higher PM_2.5_ exposure associated with higher IL-6 concentrations in females but not males. Additionally, higher PM_2.5_ concentrations were significantly associated with greater total anxiety, generalized anxiety, and social anxiety symptoms, but only in females. Higher IL-8 concentrations were associated with greater depressive symptoms, and a significant TNF-α-by-sex interaction was observed for total and social anxiety symptoms, with higher TNF-α concentrations linked to greater symptoms in females but not males.

**Discussion:**

These findings suggest that PM_2.5_ exposure is associated with inflammation and anxiety symptoms in adolescence, with notable sex differences. As a modifiable risk factor, reducing outdoor air pollution exposure may help mitigate psychiatric symptoms in youth.

## 1 Introduction

Outdoor air pollution is a significant driver of global disease burden, linked to cardiovascular and respiratory diseases, cancers, and other health conditions (1). In 2021, air pollution ranked as the second leading risk factor for mortality worldwide, exceeded only by high blood pressure. This underscores its critical impact on global health and the urgent need for targeted interventions. Among air pollutants, PM_2.5_—particulate matter with an aerodynamic diameter of less than 2.5 microns—represents the largest contributor to the global disease burden (1). PM_2.5_ is particularly concerning due to its small size, which allows it to bypass the body’s natural barriers, including the blood-brain barrier, and embed more deeply in the lungs. This can trigger systemic inflammatory responses, which may indirectly affect the central nervous system, further amplifying its adverse health effects (2–4).

While the adverse effects of PM_2.5_ on physical health are well-documented, accumulating evidence suggests that this pollutant may also significantly impact mental health. Chronic PM_2.5_ exposures are associated with worsening psychiatric disorders (e.g., anxiety and depression), as well as cognitive dysfunction, with exposure durations varying across studies (e.g., past-month, past-year) (3, 5). However, even acute elevations in air pollution (i.e., days prior) have been associated with increased emergency department visits for psychiatric symptoms in children and adults (6–8), underscoring the importance of investigating the psychological consequences of short-term increases in air pollution.

While the adverse mental health effects of PM_2.5_ have been documented across the lifespan, adolescence is a particularly vulnerable period, when the brain may be particularly sensitive to the adverse effects of PM_2.5_. During adolescence, the brain undergoes significant pruning and reorganization, particularly in frontolimbic pathways that subserve emotional processing and executive functioning (9). Additionally, youth inhale more air relative to their body size than adults, and their natural barriers—such as the blood-brain barrier and the nasal, gut, and lung epithelia—are still maturing. As a result, these immature barriers may be less effective at filtering and trapping air pollutants (10). Adolescence is also a critical period for the onset of psychiatric disorders, with mental health conditions, including anxiety and depressive disorders, typically emerging during this developmental stage (11, 12). As such, understanding the association between PM_2.5_ exposure and mental health during adolescence is crucial for identifying early intervention strategies and mitigating potential long-term effects.

Systemic inflammation is increasingly recognized as a key mechanism linking air pollutants (3), like PM_2.5_, to physical and mental health outcomes. Investigating inflammatory markers is crucial for understanding how air pollution impacts the developing brain. Exposure to PM_2.5_ can trigger inflammatory responses that disrupt neurobiological processes, further exacerbating psychiatric risk during adolescence. Previous research has demonstrated that recent exposure to air pollution (e.g., days to 1 month prior to sample), including PM_2.5_, is associated with elevated concentrations of peripheral inflammatory markers (e.g., interleukin-6 (IL-6), tumor necrosis factor alpha (TNF-α)) in children and adolescents (13–15). These elevated inflammatory markers have been linked to various adverse health outcomes, including respiratory diseases, cardiovascular conditions, and metabolic disorders (13–15). However, fewer studies examine the inflammatory pathways that may mediate effects on mental health in youth. This gap is particularly relevant given the emerging evidence suggesting that inflammation plays a central role in the development of psychopathology, including anxiety and depression (9).

A remarkably underexplored area is the role of lipid mediators in the inflammatory response associated with air pollution exposure. Pro-inflammatory (e.g., prostaglandins [PGs]) and anti-inflammatory (e.g., hydroxy-PUFAs [HETEs]) lipid mediators play a critical role in regulating inflammatory and immune responses (16). They provide unique insights into the generation and resolution phases of inflammation, which is crucial for understanding how acute and chronic exposure to air pollution may disrupt homeostasis and contribute to psychiatric risk. Both *in vivo* and *in vitro* studies have demonstrated that particulate matter exposure can induce the production of these lipid mediators, which may precipitate and/or potentiate systemic inflammatory responses (17–19). Thus, understanding the role of lipid mediators in the inflammatory response to air pollution is essential, especially during adolescence, when dynamic neuro-immune interactions influence brain maturation and behavioral regulation, potentially heightening vulnerability to environmental stressors and psychiatric risk (20).

The current exploratory study aimed to investigate the relationship between recent exposure to PM_2.5_, peripheral markers of inflammation, and anxiety and depressive symptoms in adolescents. Specifically, we focused on key peripheral inflammatory markers, including pro-inflammatory cytokines, c-reactive protein (CRP), and lipid mediators, to elucidate their role in the effects of PM_2.5_ on psychiatric risk. Additionally, given evidence of sex differences in inflammatory marker concentrations (21) and the relationship between air pollution exposure and psychiatric symptoms (3, 22), we examined whether associations between PM_2.5_, inflammation, and psychiatric symptoms differed by sex.

## 2 Materials and methods

### 2.1 Participants

Participants (adolescents and one parent/guardian) were recruited from the Detroit, Michigan, USA, metropolitan area (approximately ≤ 50 miles from the city) between 2021 and 2024. Recruitment efforts included community-based advertisements and flyers, partnerships with local schools, recreation centers, a local pediatrics practice (Wayne Pediatrics), and a children’s health center (The Children’s Center).

Eligibility criteria included children aged 10–17 years, right-handedness, and English speaking. Exclusion criteria included current or past pervasive development disorders or other severe psychopathology (e.g., schizophrenia, autism spectrum disorder), history of neurological disorder (e.g., epilepsy, traumatic brain injury), current or potential pregnancy, contraindications for phlebotomy, significant sensory impairments (e.g., hearing loss), alcohol detection with breathalyzer or positive urine drug screen, oral contraceptive use, and non-stimulant medication use. Study visits were scheduled between 10 AM and 4 PM to minimize circadian effects. Height and weight were collected to compute body mass index (BMI).

Parents/guardians provided written informed consent, and adolescents provided assent before any study procedures. This study was approved by the Wayne State University Institutional Review Board. A total of 123 adolescent-caregiver dyads enrolled in a parent study on adolescent neurodevelopment and anxiety risk. Of the 123, 99 (80.5%) provided blood samples. Of 99, 21 adolescents reported medication use (i.e., over-the-counter allergy medications, acetaminophen/ibuprofen) the day prior or the day of plasma sample collection and were excluded from analyses. Thus, the final sample for this study was 78 adolescents.

### 2.2 Air pollution estimates

Participants’ current addresses were geocoded using our previously described DeGAUSS software and geocoder (23). Daily average PM_2.5_ concentrations were estimated at the home address using a generalized random forest model via the Air Pollution Prediction Commons (appc 0.5.0) (24). This model trains weather and atmospheric data, wildfire smoke plumes, elevation, and satellite-based aerosol products on PM_2.5_ concentrations from the EPA’s Air Quality System. Daily estimates were averaged over the month before the participant completed the study as an estimate of recent past-month exposure (24). Cross-validated model accuracy for monthly concentrations is highly accurate, with a median absolute error of 0.72 µg/m³ and 0.89 correlation between predicted and observed values. A histogram of past-month PM_2.5_ concentrations in the sample is provided in **Supplementary Figure 1**.

In addition to air pollution estimates, residential addresses were also used to determine the 2020 national rank of the Area Deprivation Index (ADI) (25), a composite measure of neighborhood socioeconomic disadvantage, to be included as a covariate in all analyses. The 2020 ADI metric was selected as the most recent estimate available at the start of data collection (August 2021) to ensure it reflected socioeconomic conditions closest to participants’ enrollment without extending beyond the study period. The ADI has been used in prior research examining associations between PM2.5 and health outcomes in both youth and adult populations (26, 27). To categorize the level of neighborhood disadvantage, the ADI was dichotomized based on the >75th percentile threshold, distinguishing participants from neighborhoods in the highest and lowest quartiles of socioeconomic disadvantage.

### 2.3 Anxiety and depression symptoms

Anxiety symptoms were assessed using the child-report version of the 41-item Screen for Child Anxiety-Related Disorders (SCARED) (28). The SCARED evaluates symptoms over the past three months and provides a total score, along with subscores for specific types of anxiety: panic, generalized anxiety disorder (GAD), separation anxiety, social anxiety, and school avoidance. We focused on the total score and the GAD and social anxiety disorder subscores, as these have been linked to recent traffic-related PM_2.5_ air pollution exposure in children and adolescents (29). Depression symptoms were assessed using the Child Depression Inventory-short form (CDI-SF) (30). Sum scores were calculated, with higher scores indicating greater anxiety and depressive symptoms. The CDI total scores were positively skewed and thus were log_10_ transformed for analysis.

### 2.4 Peripheral inflammatory marker collection and analysis

Non-fasting blood samples were collected via venipuncture into ethylenediamine tetraacetic acid (EDTA) tubes (BD Vacutainer, GK3E EDTA). Samples were centrifuged at 4°C and 1,500 RPM within 5 min of collection and plasma was stored at -80 C until processing.

Following study completion, plasma samples were analyzed at the Wayne State University Lipidomics Core Facility using liquid chromatography-tandem mass spectrometry (LC-MS/MS). LC-MS/MS (QTRAP7500with SelexIon system, Sciex) methods were employed for qualitative profiling of the lipidome utilizing Information Dependent Acquisition (IDA) strategies and quantitative analysis using Multiple Reaction Monitoring (MRM) methods, following previously published protocols (31). LipidView was used for lipid identification, and MultiQuant was used to process the chromatographic data. For the current study, we focused on inflammatory lipid mediators derived from cyclooxygenase (COX) and lipoxygenase (LOX) pathways, as these are associated with short-term air pollution exposure in adults (18). These included PGE2, PGE3, 12(S)-HETE, 15(S)-HETE, and 12-HEPE.

For the pro-inflammatory cytokines (IL-6, IL-8, and TNF-α) and CRP analysis, plasma levels were quantified using the U-PLEX Proinflammatory Combo 4 (human) and Human CRP Assay according to the manufacturer’s instructions. The assays were run using a MESO QuickPlex SQ 120 instrument, and data analysis was conducted using the Discover Workbench Desktop Analysis Software (version 4.0; Meso Scale Diagnostics).

Plasma lipid mediator and pro-inflammatory cytokine concentrations were positively skewed and were log_10_ transformed to normalize the distribution. Descriptive statistics (median, IQR, range) for the raw values of the inflammatory markers are provided in **Supplementary Table S1**.

### 2.5 Selection of covariates and statistical analysis

Statistical analysis and data visualization were performed using R (version 4.4.2) with R Studio (version 2024.09.1). Descriptive statistics were used to characterize the study sample demographics, psychiatric symptoms, and inflammatory markers. Age, biological sex, BMI, the 2020 national rank of Area Deprivation Index (ADI) with a cutoff at the 75^th^ percentile, and parental smoking in the home were selected as potential covariates based on prior research indicating associations with either PM_2.5_, inflammatory markers, or psychiatric symptoms (21, 32–34). While PM_2.5_ is only significantly correlated with sex and ADI in the sample, we included additional covariates to increase the precision of our estimates, given their known associations with variability in psychiatric symptoms (age sex), and inflammatory markers (BMI, parental smoking). These covariates were included in analyses where applicable. Spearman correlations were calculated to assess the relationships between PM_2.5_ concentrations, inflammatory markers, psychiatric symptoms, and covariates. The full correlation matrix for these variables can be found in **Supplementary Tables S2-S4**. Since puberty may be associated with changes in inflammatory markers (21), supplemental analyses were also performed using pubertal status instead of age as a covariate, and these analyses are described in the **Supplementary Material**.

### 2.6 Interaction terms and model specification

Linear regression analyses were conducted to assess the associations between past-month PM_2.5_ concentrations and inflammatory markers and between PM_2.5_ and psychiatric symptom scores.

Sex differences were considered in all analyses, given the well-documented evidence that both inflammatory markers (21) and psychiatric symptoms vary by sex (35, 36). However, it remains unclear whether PM_2.5_’s effects on inflammation or psychiatric symptoms differ by sex, warranting the inclusion of interaction terms to explore these potential moderating effects.

For inflammatory markers, the main effect of sex and the PM_2.5_*sex interaction term were included to assess whether PM_2.5_-related inflammation differed by sex. Similarly, in the psychiatric symptom analyses, an interaction term between PM_2.5_ exposure and sex was included based on prior literature suggesting that air pollution effects on mental health outcomes vary by sex (3, 22). Additionally, in this sample, females had both higher PM_2.5_ exposure and higher levels of anxiety symptoms compared to males. The main effect of sex was omitted from the psychiatric symptom models to prevent overfitting and to isolate the effect of PM_2.5_ within each sex group.

Lastly, the analysis of inflammatory markers in relation to psychiatric symptoms also included both main effects and an interaction term to assess whether inflammation-psychiatric symptom associations differed by sex, given the lack of clarity in existing research.

## 3 Results

### 3.1 Demographics

The sample was 48.7% female, with the mean age of 13.3 years (range: 10-17). The sample was racially and ethnically diverse (36% White Non-Hispanic, 33% Black Non-Hispanic, 15% Hispanic, 7% More Than One Race, 1% Other), and 45% and 55% of youth had clinically significant anxiety and depressive symptoms, respectively. The mean past-month PM_2.5_ concentration was 9.11 ± 2.68 µg/m³, ranging from 5.78 to 16.83 µg/m³. Sex differences were observed in several demographics: females had higher mean past-month PM_2.5_ concentrations (9.79 + 2.68 µg/m^3^) than males. Additionally, a higher percentage of females (68%) had clinically significant anxiety symptoms compared to males (23%). Females also had a higher mean BMI than males (25.11 + 5.17 kg/m^2^). Further demographic details are provided in **Table 1**.

**Table 1:** Participant characteristics (N=78).

Demographic	Full Sample (N=78)	Males (N=40)	Females (N=38)	Males vs. Females comparisons
	N (%) or Mean ± SD			P-value
Age	13.28 ± 2.28	13.10 + 2.05	13.47 + 2.51	p = 0.473
Body Mass Index (BMI)	23.51 ± 4.94	22.02 + 4.28	25.11 + 5.17	**p = 0.005**
Race/Ethnicity				p = 0.203
White, non-Hispanic	28 (35.9)	16 (40.0)	12 (31.6)	
Black, non-Hispanic	26 (33.3)	10 (25.0)	16 (42.1)	
Hispanic	12 (15.4)	5 (12.5)	7 (18.4)	
More than one race	6 (7.7)	5 (12.5)	1 (2.6)	
Other	1 (1.3)	0 (0)	1 (2.6)	
Household Income				p = 0.217
Low (< $50,000)	33 (42.3)	14 (35.0)	19 (50.0)	
Middle ($50,000 - $100,000)	17 (21.8)	8 (20.0)	9 (23.7)	
High (> $100,000)	28 (35.9)	18 (45.0)	10 (26.3)	
Pubertal Status				**p = 0.028**
Pre-puberty	11 (14.1)	9 (23.7)	2 (5.6)	
Post-puberty	63 (80.8)	29 (76.3)	34 (94.4)	
Smokers in Home	10 (12.8)	4 (10.0)	6 (15.8)	p = 0.512
Met or Exceeded Clinical Cutoff for Anxiety	35 (44.9)	9 (22.5)	26 (68.4)	**p < 0.001**
Met or Exceeded Clinical Cutoff for Depression	43 (55.1)	19 (47.5)	24 (63.2)	p = 0.253
Total SCARED Score	25.26 ± 15.37	17.72 + 10.21	33.00 + 16.03	**p < 0.001**
Total CDI Score	3.91 ± 3.81	3.13 + 3.47	4.71 + 4.01	p = 0.068
Past-month PM_2.5_ concentration (ug/m^3^)	9.11 ± 2.68	8.47 + 2.55	9.79 + 2.68	**p = 0.029**
2020 National Rank of Area Deprivation Index (ADI) 75th Percentile Cutoff	35 (44.9)	12 (31.6)	23 (62.2)	**p = 0.008**

Anxiety symptoms were measured using the cutoff score (≥25) on the Screen for child Anxiety-Related Disorders (SCARED). Depression symptoms were measured using the cutoff score (≥3) on the Children's Depression Inventory (CDI) Short Form. Smoking in Home was measured via parental self-report. Differences in demographic characteristics between males and females were tested using independent sample t-tests for continuous variables (e.g., age) and chi-squared tests for categorical variables (e.g., parental smoking in home). Pubertal status was measured using child self-report of Tanner Stages.

Bolded values indicate p < 0.05.

### 3.2 PM_2.5_ and inflammatory lipid mediators, cytokines, and CRP

**Figure 1:**
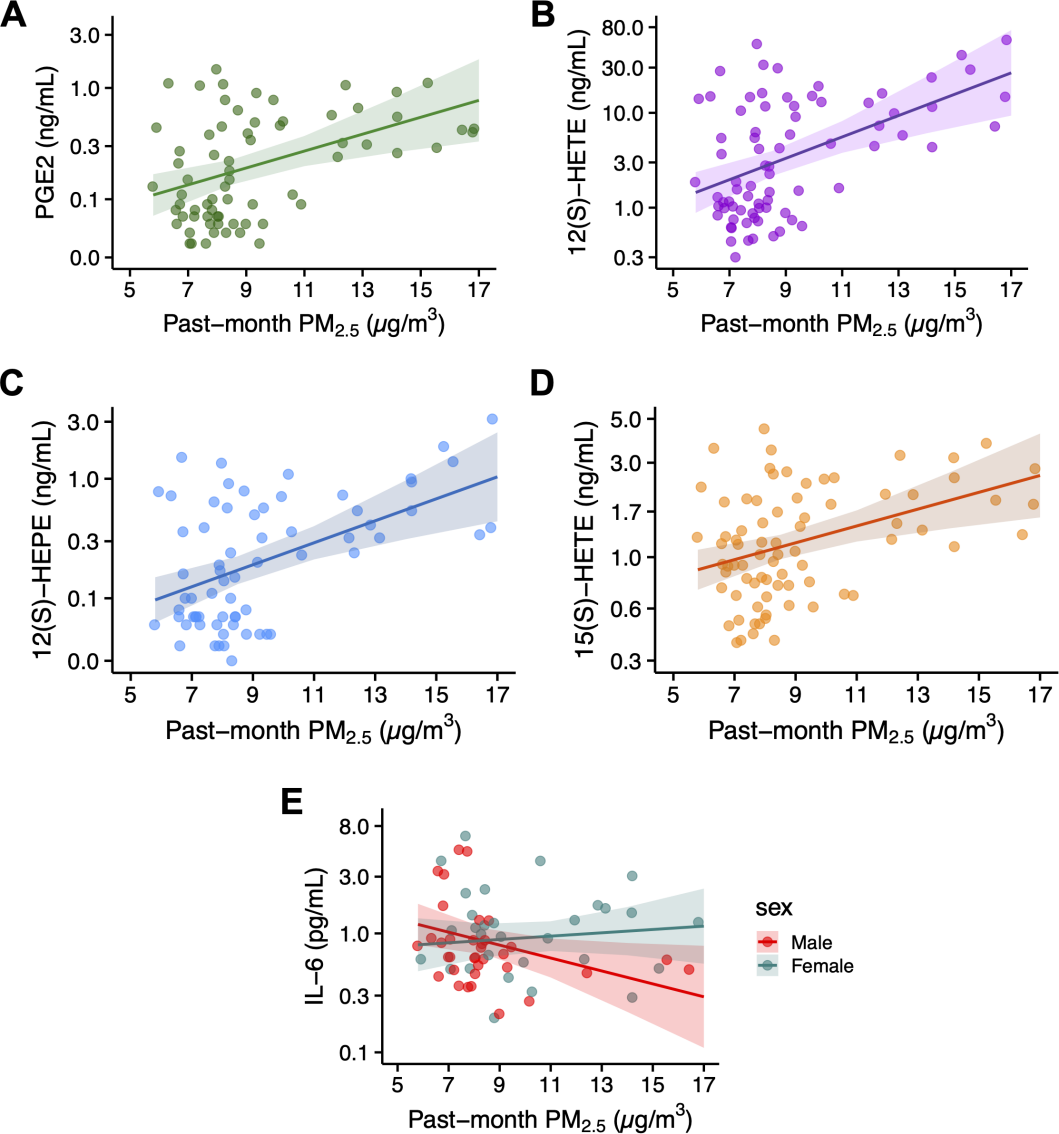
Relationship between past-month PM_2.5_ concentrations and peripheral inflammatory markers. Panels A-D display associations between PM_2.5_ and lipid mediators: **(A)** PGE2, **(B)** 12(S)-HETE, **(C)** 12(S)-HEPE, and **(D)** 15(S)-HETE. Panel **(E)** illustrates the interaction between PM2.5 and biological sex on IL-6 concentrations, with regression lines fitted separately for each sex (indicated by color). In all panels, regression lines are fitted to the data, with shaded areas representing the 95% confidence intervals. The y-axis is presented on a logarithmic scale to facilitate visualization across multiple orders of magnitude.

Past-month PM_2.5_ concentrations were significantly associated with concentrations of PGE2, 12(S)-HETE, 12(S)-HEPE, and 15(S)-HETE. Specifically, for each 1 µg/m^3^ increase in PM_2.5_, PGE2 levels increased by 20% (β = 1.20). Similar significant increases in concentrations were observed for 12(S)-HETE (29%), 12(S)-HEPE (23%), and 15(S)-HETE (10%). These results are visually displayed in **Figures 1A–D**). No significant associations were observed between past-month PM_2.5_ and CRP, IL-8, or TNF-α (p’s > 0.05, see **Supplementary Figure 2**). Past-month PM_2.5_ concentrations were significantly associated with IL-6 concentrations, with a differential effect by sex. For each 1 µg/m³ increase in PM_2.5_, IL-6 levels decreased by 11% in males and increased by 4% in females (see **Figure 1E**). There was a main effect of sex on PGE3 levels, with females exhibiting higher PGE3 concentrations than males (β = 1.55), but no significant association was observed between past-month PM_2.5_ and PGE3 (p = 0.65). No other significant interactions between PM_2.5_ and sex were observed for the other inflammatory markers. The model results for these analyses can be found in **Supplementary Tables S5, S6**.

### 3.3 PM_2.5_ and psychiatric symptoms

**Figure 2:**
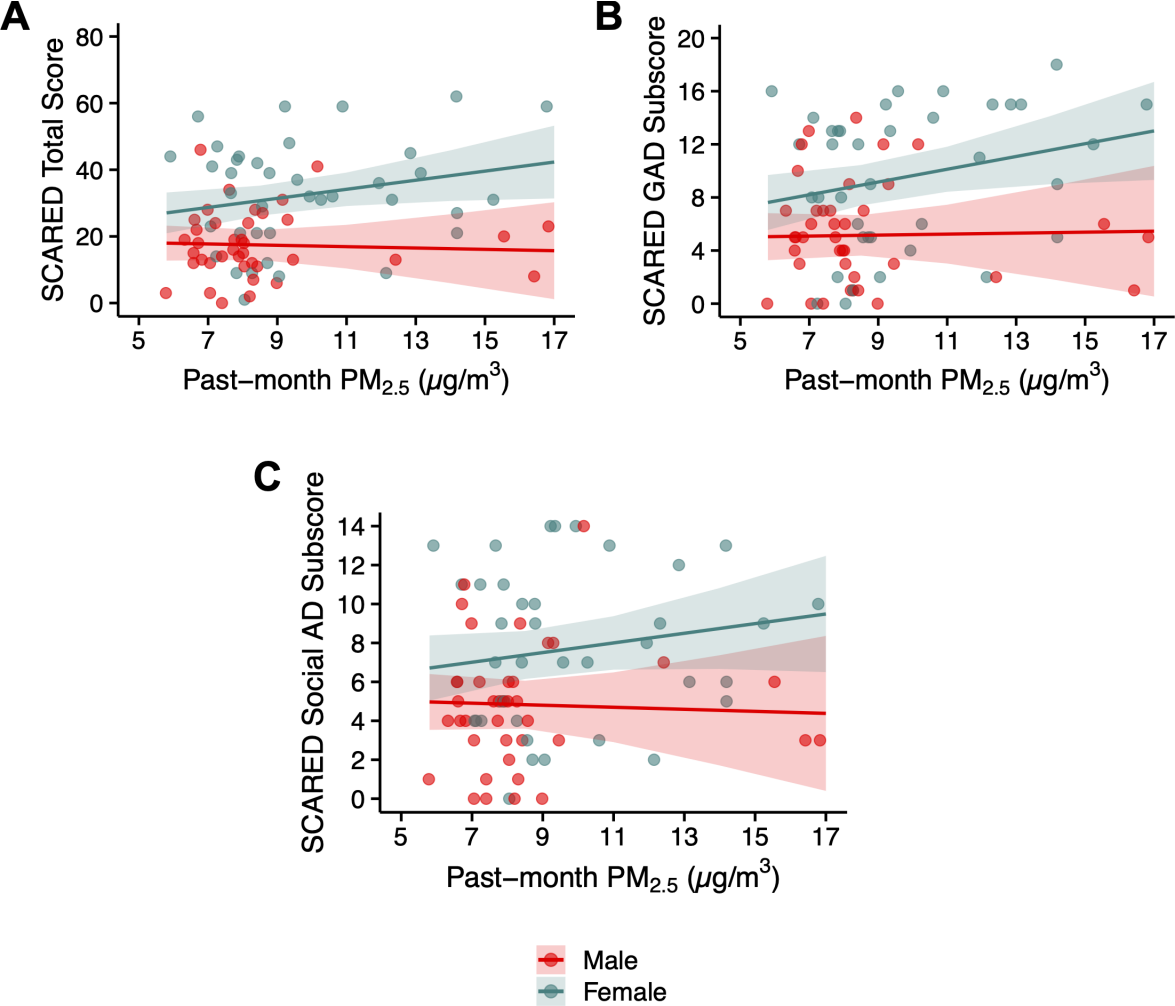
Relationship between past-month PM_2.5_ concentrations and anxiety symptoms. **(A)** SCARED Total Score, **(B)** SCARED Generalized Anxiety Disorder (GAD) Subscore, **(C)**: SCARED Social Anxiety Disorder (AD) Subscore. In each panel, the regression lines are fitted to the data for each sex group (indicated by color). The shaded area represents the 95% confidence intervals of the regression estimates.

Past-month PM_2.5_ concentrations were significantly associated with mental health symptoms, with differential effects observed by sex. Significant interaction between PM_2.5_ and sex were found for the SCARED total score, GAD subscore, and Social AD subscore. These results are visually displayed in **Figures 2A–C**. Specifically, for each 1 µg/m³ increase in PM_2.5_, SCARED total scores increased by 1.36 in females, with no significant effect observed for males. Similarly, the GAD subscore increased by 0.48 in females per 1 µg/m³ increase in PM_2.5,_ with no significant effect in males, and the Social AD subscore increased by 0.25 in females with no significant effect in males. No significant associations were observed between past-month PM_2.5_ and the depression total score (p > 0.05). The model results for these analyses can be found in **Supplementary Table S7**.

### 3.4 Inflammatory markers and psychiatric symptoms

**Figure 3:**
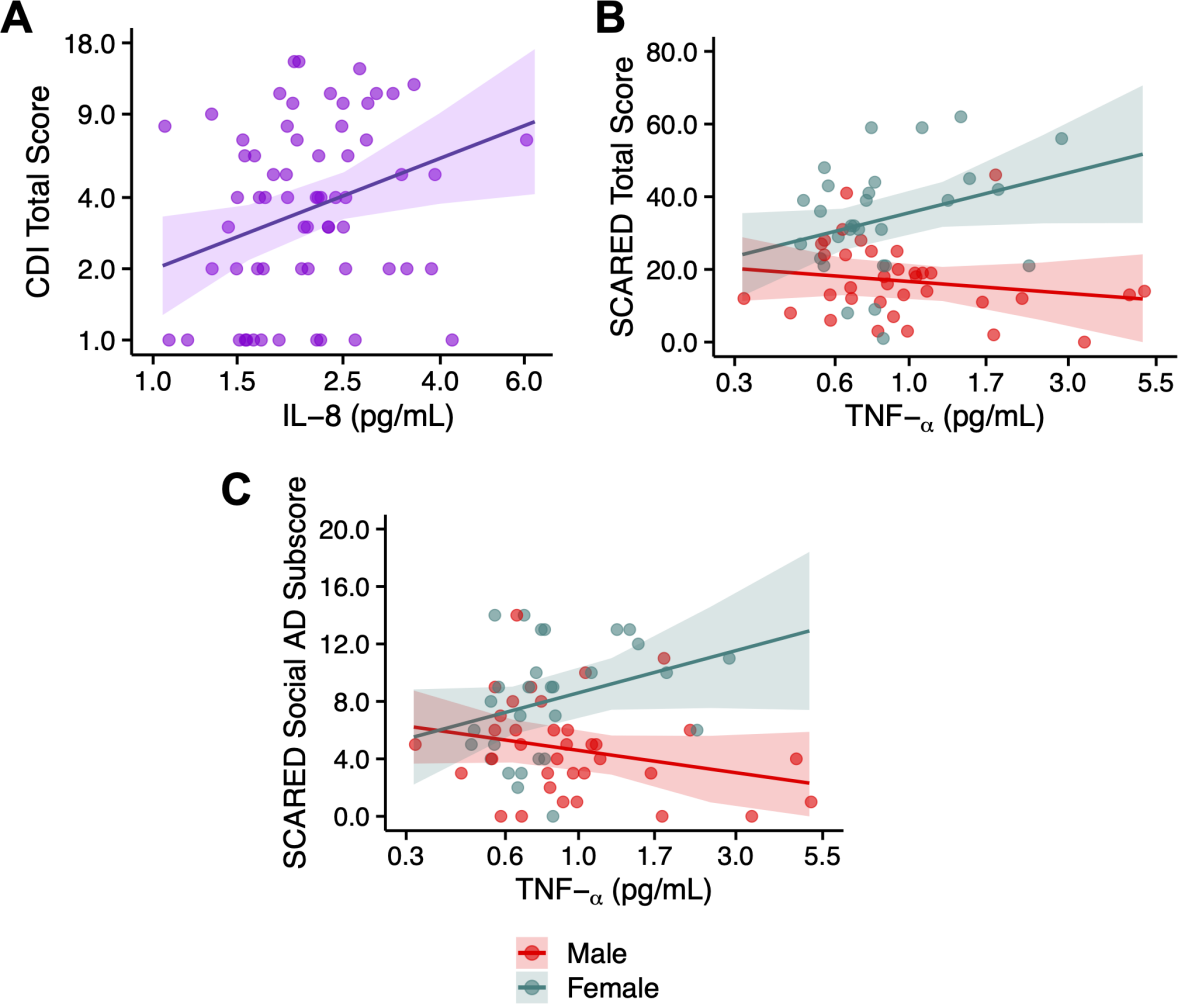
Relationship between inflammatory markers and psychiatric symptoms. **(A)** IL-8 and Child Depression Inventory (CDI) Total Score, **(B)** TNF-α and SCARED Total Score, **(C)** TNF-α and SCARED Social Anxiety Disorder(AD) Subscore. In panels **(B, C)**, the regression lines are fitted to the data for each sex group (indicated by color). The shaded area represents the 95% confidence intervals of the regression estimates. The x-axis is presented on a logarithmic scale to facilitate visualization across multiple orders of magnitude.

A significant main effect of IL-8 was observed for the CDI total score, with each 10-fold increase in IL-8 associated with a 6.03-fold increase in the CDI total score. Significant interactions between TNF-α levels and sex were found for the SCARED total score and SCARED Social AD subscore. For the SCARED total score, higher TNF-α levels were associated with an increase for females (β = 22.92), with no significant effect observed in males. Similarly, for the SCARED Social AD subscore, higher TNF-α levels were linked to an increase for females (β = 6.09), with no significant effect for males. Additionally, a main effect of sex was observed across all inflammatory models, with females generally exhibiting higher anxiety and depression symptoms than males. No significant associations were observed for PGE2, PGE3, 12(S)-HETE, 12(S)HEPE, 15(S)HETE, IL-6, or CRP with psychiatric symptoms. These results are presented in, **Figure 3** and **Supplementary Tables S8–S16**.

## 4 Discussion

To our knowledge, this is the first study to investigate the relationships between recent PM_2.5_ exposure, peripheral inflammatory markers, and psychiatric symptoms in adolescents. We found that adolescents with higher past-month PM_2.5_ concentrations had significantly higher inflammatory markers, specifically PGE2 (prostaglandin E2), 12(S)-HETE (12-hydroxy-eicosatetraenoic acid), 12(S)-HEPE (12-hydroxy-eicosapentaenoic acid), and 15(S)-HETE (15-hydroxy-eicosatetraenoic acid). Additionally, we observed a significant main effect of sex on PGE3, with females exhibiting higher concentrations than males; however, since PGE3 was not associated with PM_2.5_ exposure or psychiatric symptoms, its role in linking air pollution to psychiatric symptoms appears limited. Of note, the past-month PM_2.5_ concentrations observed in this study ranged from 5.78 to 16.83 µg/m³. While the EPA’s standards for PM_2.5_ are set at an annual average of 9 µg/m³ (EPA, 2024), two-thirds of our sample had exposures below this threshold, indicating that most participants experienced levels considered to be lower risk. Despite this, we observed significant associations with inflammatory markers, suggesting that even lower levels of PM_2.5_ exposure may contribute to adverse health effects in adolescents.

Our findings reveal that PM_2.5_ exposure is associated with alterations in lipid mediators involved in inflammatory responses, including PGE2, 12(S)-HETE, 12(S)-HEPE and 15(S)-HETE. PGE2 is derived from the COX (cyclooxygenase) pathway and plays a complex role in inflammation. Further, PGE2 acts as a key initiator of the early inflammatory response and also contributes to resolution in later phases (37). In contrast, 12(S)-HETE, 12(S)-HEPE, and 15(S)-HETE originate from the 5-LOX (5-lipoxygenase) pathway (18), which is involved in resolving acute inflammation. The LOX-derived lipid mediators are widely expressed in the cardiovascular and central nervous systems, with localization within the primary olfactory cortex and hippocampus (38, 39).

Similar – lipid mediator profiles have been observed in prior studies of air pollution. Anti-inflammatory lipid mediators, including 12(S)-HETE, 12(S)-HEPE, and 15(S)-HETE have been shown to increase in lower animals and in adults following recent/acute (e.g., days to months prior) air pollution exposure (18, 19, 40). Conversely, significant decreases of 12(S)-HETE, 12(S)-HEPE, and 15(S)-HETE were reported when air pollution decreased during the 2018 Beijing, China Olympics (41, 42). Interestingly, a recent study in adults observed a spike in urinary PGE2 concentrations 8 hours after exposure to a wildfire event ceased, which is characterized by drastic increases in PM_2.5_ concentrations due to smoke (43). However, the exact functional role of this increase – whether pro-inflammatory or contributing to inflammation resolution – remains unclear (43), highlighting the complex and context-dependent role of PGE2 in the inflammatory process, particularly in the context of air pollution exposure.

To our knowledge, this is the first study to report elevations of specific lipid mediators associated with air pollution in adolescents, highlighting the need to understand the developmental impacts of air pollution on the immune and inflammatory systems. Such a disruption in the equilibrium between anti- and pro-inflammatory mediators (referred to as “unalamation”) may contribute to chronic low-grade inflammation and disease risk (44). Notably, despite these associations, we did not observe significant associations between PM_2.5_ exposure and canonical pro-inflammatory markers, such as CRP and TNF-α. This contrasts with three prior studies linking PM_2.5_ exposure to increased levels of these markers in children and adolescents (13, 14). However, two of these studies were conducted outside the US, potentially involving different PM_2.5_ compositions, and all three focused on narrower age ranges—specifically, two studies included younger children (8–11 years old) (13–15), and the third only included 17-year-olds (15). In contrast, our study specifically examined a broader adolescent age range (10–17 years), a developmental stage marked by significant biological and physiological changes. Differences in exposure windows and biological measures (e.g., plasma vs. exhaled breath) may also account for these discrepancies.

However, we did find a significant PM_2.5_-by-sex interaction on IL-6 concentrations. In females, increased PM_2.5_ exposure was associated with higher IL-6 concentrations, whereas in males, the relationship was reversed, with lower IL-6 concentrations. These divergent patterns suggest that inflammatory responses to PM_2.5_ may differ between sexes, potentially reflecting variation in immunoregulatory mechanisms and hormonal influences that may shape cytokine production (20, 21). While the clinical implications of higher versus lower IL-6 concentrations remains to be fully understood, these findings underscore the complexity of the inflammatory response to environmental pollutants.

We also found that adolescents with higher past-month PM_2.5_ exposure had significantly more total, generalized anxiety, and social anxiety symptoms, highlighting a potential link between air pollution exposure and mental health in adolescents - a population uniquely vulnerable to environmental stressors. Importantly, our PM_2.5_ by sex interaction analyses revealed that these associations were driven primarily by females. In our sample, females not only exhibited significantly higher anxiety symptoms in general but also had higher past-month PM_2.5_ concentrations. PM_2.5_ exposure was assessed via residential address, indicating that these exposure differences are not due to personal characteristics per se but rather reflect the fact that, in our sample, females tended to reside in more deprived neighborhoods that likely have higher outdoor PM_2.5_ concentrations.

These findings raise an important question: Could reducing exposure to fine particulate matter decrease the burden of anxiety disorders in youth? Our findings are also consistent with our earlier longitudinal study of unmedicated adolescents with GAD in which we observed that daily fluctuations in ambient PM_2.5_ concentrations predicted changes in anxiety symptom severity two to four days after exposure (45). Taken together, these data suggest that air pollution not only correlates with anxiety symptoms but may also actively contribute to their persistence and exacerbation over time. This finding highlights the need for greater awareness of environmental risk factors in anxiety disorders and underscores the potential clinical value of integrating air quality considerations into prevention and intervention strategies.

Furthermore, significant interactions between TNF-α concentrations and sex were found for both total anxiety and social anxiety symptoms. Specifically, higher TNF-α was associated with higher anxiety symptoms in females only. These findings are consistent with recent work in adults, where elevated TNF-α concentrations were not only significantly higher in those with GAD but also positively correlated with anxiety symptoms (46). Moreover, a recent preclinical study has demonstrated that sustained TNF signaling is associated with the development of anxiety-like behaviors (47). Together, these studies support the notion that TNF-α may play a critical role in the pathophysiology of anxiety, underscoring the importance of further investigating inflammatory pathways as potential targets for intervention.

Beyond its associations with anxiety, higher IL-8 concentrations were also significantly linked to increased depressive symptoms. This finding aligns with a recent study in a community-based sample of adolescents aged 13–19, which similarly reported an association between elevated IL-8 levels and greater depressive symptoms (48). Taken together, these findings suggest that specific inflammatory pathways may differentially contribute to distinct psychiatric outcomes in adolescence.

Our findings have significant public health implications. PM_2.5_ may represent a novel and modifiable exposure and an actionable and measurable target for interventions that could reduce psychiatric morbidity on a population level. This is particularly important given that anxiety symptoms and anxiety disorders in this population are associated with significant morbidity including academic underachievement, increased risk of mood disorders, suicidality and impairment in peer and family relationships. As such, by addressing environmental risk factors such as air pollution (49), we could potentially mitigate the prevalence and severity of anxiety disorders in children and adolescents, shifting the focus from reactive treatment to proactive prevention.

While this study provides valuable initial insights into the relationship between air pollution exposure and peripheral markers of inflammation—potentially relevant to the risk of psychopathology—several limitations warrant discussion. One key limitation is that inflammatory markers were assessed at a single timepoint, which prevents the evaluation of changes in these markers over time. Next, PM_2.5_ exposure was averaged over the past month, which may obscure acute fluctuations due to its high temporal variability (50). Future studies should assess inflammatory markers at multiple time points and consider exposure windows that better capture short-term fluctuations. Additionally, children’s inflammatory systems may become more sensitive to challenges or stresses following PM_2.5_ exposure (51). To better capture these dynamic responses, future research should incorporate stress-inducing tasks to examine changes in real-time inflammatory responses in those exposed to higher concentrations of air pollution. Further, participants were primarily drawn from the Detroit, MI area, which limits the generalizability of these findings. Next, our study focuses on a specific period, late childhood through adolescence. Importantly, while anxiety symptoms may begin earlier, this is the period of maximal increases in anxiety symptoms and the emergence of anxiety disorders. Thus, it remains possible that the effects of PM_2.5_ on symptoms may emerge later in development or interact with other risk factors over time, which could contribute to variability in findings across studies. Further, self-report measures have inherent limitations and may differ from symptoms assessed by clinicians. Finally, whether these symptoms represent psychiatric disorders (e.g., GAD) cannot be determined in this sample as we did not include assessment by a clinician.

In conclusion, this exploratory study represents a critical first step in understanding the complex interplay between air pollution exposure, systemic inflammation, and mental health in adolescents. To our knowledge, this is the first study to examine these relationships during adolescence, a sensitive period for neurodevelopment and heightened psychiatric risk. Our findings suggest that youth with higher recent PM_2.5_ exposure have elevated concentrations of anti-inflammatory lipid mediators and anxiety symptoms, highlighting the need for further research into the pathways linking environmental exposures to mental health outcomes.

## 5 Conflict of interest

Dr. Strawn has received research support from the National Institutes of Health NIMH/NCATS/NIEHS/NICHD, Patient-Centered Outcomes Research Institute PCORI, MindMed and material support from Myriad Genetics. He receives royalties from Springer and Cambridge University Press, honoraria from the Neuroscience Education Institute, and is an author for UpToDate. He has consulted to MindMed, Abbvie Cerevel, Otsuka, and Genomind. All other co-authors have no conflicts of interest to disclose.

## Data Availability

The datasets presented in this article are not readily available because they are not publicly shared. However, data may be made available upon reasonable request to the corresponding and senior authors for collaboration or further analysis. Requests to access the datasets should be directed to Clara Zundel, clara.zundel@wayne.edu and Hilary Marusak, hmarusak@med.wayne.edu.
