# Supplementary material for: Outdoor air pollution and psychiatric symptoms in adolescents: a study of peripheral inflammatory marker associations
Source: Front Psychiatry. 2025 May 23;16:1588964. doi: 10.3389/fpsyt.2025.1588964 (PMC12143268; doi:10.3389/fpsyt.2025.1588964)
Supplement: Supplementary file 1 [file DataSheet1.docx]

Supplementary Material

# Supplementary Figures

**Supplemental Figure 1.** Distribution of past-month PM_2.5_ levels (µg/m³) among the sample. The x-axis represents PM_2.5_ concentrations, while the y-axis represents the frequency of occurrences.

**Supplemental Figure 2.** Non-significant relationships between past-month PM_2.5_ concentrations and peripheral inflammatory markers. A: PGE3, B: IL-8, C: TNF-α, D: CRP. In all panels, the y-axis is presented on a logarithmic scale to improve the visualization of data across multiple orders of magnitude.

**Supplemental Figure 3.** Model diagnostic plots for significant analyses of the relationship between past-month PM_2.5_ concentrations and peripheral inflammatory markers. **Panel A)** PGE2, **Panel B)** 12(S)-HETE, **Panel C)** 12(S)-HEPE, **Panel D)** 15(S)-HETE, **Panel E)** IL-6.

**Supplemental Figure 4.** Model diagnostic plots for significant analyses of the relationship between past-month PM_2.5_ concentrations and anxiety symptoms. **Panel A**: SCARED Total Score, **Panel B**: SCARED GAD Subscore, **Panel C**: SCARED Social AD Subscore.

**Supplemental Figure 5.** Model diagnostic plots for significant analyses of the relationships between inflammatory markers and psychiatric symptoms**. Panel A**: IL-8 and CDI Total Score, **Panel B**: TNF-α and SCARED Total Score, **Panel C**: TNF-α and SCARED Social Anxiety Disorder(AD) Subscore.

# Supplementary Tables

# Supplementary tables, including descriptive statistics for the inflammatory markers, Pearson R correlations between PM_2.5_, variables of interest, and covariates, as well as the model tables, can be found in the Supplemental Tables file.

# Pubertal Status versus Age as a Covariate

In the primary analyses, age was used as a covariate to account for potential developmental effects. However, we also conducted supplementary analyses to assess whether the use of pubertal status, categorized as pre-puberty versus post-puberty, might yield different results. Pubertal status was determined based on child self-report of Tanner stages. Stages 1-2 were considered to be pre-puberty and stages 3-5 were considered post-puberty.

When pubertal status was included as a covariate instead of age, some results from the primary analyses were no longer statistically significant. Specifically, the following associations were attenuated: (1) the overall model for the effect of PM_2.5_ on 15(S)-HETE concentrations became non-significant (F(6,62) = 1.82, p = 0.11), although PM_2.5_ remained a significant predictor within the model (p = 0.01) , (2) the overall model for the effect of PM_2.5_ on the SCARED social anxiety disorder subscore became non-significant (F(6,63) = 1.81, p =0.11), although the PM_2.5_ by sex interaction remained a significant predictor within the model (p < 0.01), and (3) the TNF by sex interaction on the SCARED total score (p=0.07). These findings suggest that the inclusion of pubertal status, rather than chronological age, may attenuate some of the observed relationships between air pollution exposure, inflammatory markers, and psychiatric symptoms. It is possible that some of these attenuations may also be due to limitations in statistical power, as the sample size (N=78) may not be sufficient to detect these effects. Future research should include larger sample sizes and longitudinal designs to better capture the changes that occur across puberty.

# Social Vulnerability Index as a Covariate

In the primary analyses, the 2020 National Rank of Area Deprivation Index (ADI) was used as a covariate to account for neighborhood-level socioeconomic disadvantage. To assess whether an alternative measure might yield different findings, we conducted supplemental analyses substituting the CDC/ATSDR Social Vulnerability Index (SVI), which captures broader dimensions of community vulnerability including socioeconomic status, household composition, minority status, housing type, and transportation access.

Similar to the ADI, the SVI was dichotomized at the 75^th^ percentile to identify participants residing in areas of high social vulnerability. When SVI was included as a covariate in place of ADI, results were largely consisted with the primary analyses. The only notable change was a reduction in model significant for the association between IL-8 concentrations and depressive symptoms, which became non-significant (F(6,56) = 2.09, p = 0.07), although IL-8 remained a significant individual predictor (p = 0.03).
