# Supplementary material for: Outdoor air pollution and psychiatric symptoms in adolescents: a study of peripheral inflammatory marker associations
Source: Front Psychiatry. 2025 May 23;16:1588964. doi: 10.3389/fpsyt.2025.1588964 (PMC12143268; doi:10.3389/fpsyt.2025.1588964)
Supplement: Supplementary file 2 [file Table1.docx]

**Supplemental Tables**

| **Table S1. Inflammatory Marker Descriptive Statistics** | | | |
| --- | --- | --- | --- |
| **Inflammatory Marker** | **Median** | **IQR** | **Range** |
| PGE2 (ng/mL) | 0.18 | 0.49 | 0.04 - 1.47 |
| PGE3 (ng/mL) | 0.12 | 0.12 | 0.03 - 0.63 |
| 12(S)-HETE (ng/mL) | 2.78 | 12.76 | 0.30 - 58.76 |
| 12(S)-HEPE (ng/mL) | 0.19 | 0.50 | 0.03 - 3.16 |
| 15(S)-HETE (ng/mL) | 1.17 | 1.58 | 0.37 - 4.45 |
| IL-6 (pg/mL) | 0.78 | 0.73 | 0.20 - 5.05 |
| IL-8 (pg/mL) | 2.09 | 0.82 | 1.06 - 6.07 |
| TNF-α (pg/mL) | 0.81 | 0.46 | 0.32 - 5.07 |
| CRP (mg/L) | 0.22 | 0.77 | 0.03 - 6.00 |

| **Table S2. Spearman Correlation Coefficients Between Study Variables of Interest** | | | | | | | | | | | | | | | | | | |
| --- | --- | --- | --- | --- | --- | --- | --- | --- | --- | --- | --- | --- | --- | --- | --- | --- | --- | --- |
|  | 1 | 2 | 3 | 4 | 5 | 6 | 7 | 8 | 9 | 10 | 11 | 12 | 13 | 14 | 15 | 16 | 17 | 18 |
| **Variables of Interest** | | | | | | | | | | | | | | | | | | |
| 1. Past Month PM_2.5_ (µg/m^3^) |  | -0.135 | 0.114 | **-.317^*^** | 0.063 | **.391^**^** | -0.019 | **.429^**^** | **.388^**^** | **.343^**^** | 0.195 | 0.169 | 0.159 | 0.147 | **.237^*^** | 0.217 | 0.160 | 0.174 |
| 2. Log_10_(IL-6 pg/mL) | -0.135 |  | 0.133 | **.409^**^** | **.393^**^** | 0.072 | **.331^**^** | 0.088 | 0.052 | -0.006 | 0.146 | 0.205 | -0.012 | 0.205 | 0.090 | 0.157 | 0.007 | 0.184 |
| 3. Log_10_(IL-8 pg/mL) | 0.114 | 0.133 |  | **.268^*^** | 0.033 | **.327^*^** | 0.097 | **.354^**^** | 0.205 | 0.194 | 0.078 | 0.039 | 0.125 | 0.232 | 0.081 | -0.002 | 0.119 | 0.155 |
| 4. Log_10_(TNF-α pg/mL) | **-.317^*^** | **.409^**^** | **.268^*^** |  | -0.140 | -0.119 | 0.063 | -0.067 | -0.149 | -0.145 | -0.128 | -0.126 | -0.039 | 0.028 | -0.212 | -0.161 | 0.025 | 0.023 |
| 5. Log_10_(CRP mg/L) | 0.063 | **.393^**^** | 0.033 | -0.140 |  | 0.118 | 0.200 | 0.129 | 0.129 | 0.196 | 0.169 | 0.184 | 0.020 | 0.142 | 0.198 | 0.241 | 0.038 | 0.246 |
| 6. Log_10_(PGE2 pg/mL) | **.391^**^** | 0.072 | **.327^*^** | -0.119 | 0.118 |  | 0.142 | **.875^**^** | **.846^**^** | **.878^**^** | 0.162 | 0.154 | 0.190 | 0.008 | 0.133 | 0.211 | 0.213 | 0.015 |
| 7. Log_10_(PGE3 pg/mL) | -0.019 | **.331^**^** | 0.097 | 0.063 | 0.200 | 0.142 |  | 0.099 | 0.144 | 0.188 | **.275^*^** | 0.194 | 0.223 | 0.227 | **.265^*^** | 0.144 | 0.189 | 0.200 |
| 8. Log_10_(12(S)-HETE pg/mL) | **.429^**^** | 0.088 | **.354^**^** | -0.067 | 0.129 | **.875^**^** | 0.099 |  | **.911^**^** | **.866^**^** | 0.101 | 0.116 | 0.098 | 0.013 | 0.041 | 0.163 | 0.115 | 0.024 |
| 9. Log_10_(12(S)-HEPE pg/mL) | **.388^**^** | 0.052 | 0.205 | -0.149 | 0.129 | **.846^**^** | 0.144 | **.911^**^** |  | **.834^**^** | 0.208 | 0.203 | 0.129 | 0.121 | 0.091 | 0.204 | 0.132 | 0.144 |
| 10. Log_10_(15(S)-HETE pg/mL) | **.343^**^** | -0.006 | 0.194 | -0.145 | 0.196 | **.878^**^** | 0.188 | **.866^**^** | **.834^**^** |  | 0.106 | 0.113 | 0.107 | 0.041 | 0.081 | 0.156 | 0.137 | 0.067 |
| 11. SCARED Total Score | 0.195 | 0.146 | 0.078 | -0.128 | 0.169 | 0.162 | **.275^*^** | 0.101 | 0.208 | 0.106 |  | **.892^**^** | **.759^**^** | **.655^**^** | **.863^**^** | **.764^**^** | **.638^**^** | **.547^**^** |
| 12. SCARED Generalized Anxiety Disorder (GAD) Total Score | 0.169 | 0.205 | 0.039 | -0.126 | 0.184 | 0.154 | 0.194 | 0.116 | 0.203 | 0.113 | **.892^**^** |  | **.635^**^** | **.656^**^** | **.771^**^** | **.847^**^** | **.544^**^** | **.507^**^** |
| 13. SCARED Social Anxiety Disorder Total Score | 0.159 | -0.012 | 0.125 | -0.039 | 0.020 | 0.190 | 0.223 | 0.098 | 0.129 | 0.107 | **.759^**^** | **.635^**^** |  | **.492^**^** | **.618^**^** | **.636^**^** | **.822^**^** | **.362^**^** |
| 14. Log_10_(CDI Total Score) | 0.147 | 0.205 | 0.232 | 0.028 | 0.142 | 0.008 | 0.227 | 0.013 | 0.121 | 0.041 | **.655^**^** | **.656^**^** | **.492^**^** |  | **.611^**^** | **.591^**^** | **.424^**^** | **.867^**^** |
| 15. SCARED Total Clinical Cutoff | **.237^*^** | 0.090 | 0.081 | -0.212 | 0.198 | 0.133 | **.265^*^** | 0.041 | 0.091 | 0.081 | **.863^**^** | **.771^**^** | **.618^**^** | **.611^**^** |  | **.768^**^** | **.617^**^** | **.549^**^** |
| 16. SCARED Generalized Anxiety Disorder (GAD) Clinical Cutoff | 0.217 | 0.157 | -0.002 | -0.161 | 0.241 | 0.211 | 0.144 | 0.163 | 0.204 | 0.156 | **.764^**^** | **.847^**^** | **.636^**^** | **.591^**^** | **.768^**^** |  | **.668^**^** | **.496^**^** |
| 17. SCARED Social Anxiety Disorder Clinical Cutoff | 0.160 | 0.007 | 0.119 | 0.025 | 0.038 | 0.213 | 0.189 | 0.115 | 0.132 | 0.137 | **.638^**^** | **.544^**^** | **.822^**^** | **.424^**^** | **.617^**^** | **.668^**^** |  | **.303^**^** |
| 18. CDI Clinical Cutoff | 0.174 | 0.184 | 0.155 | 0.023 | 0.246 | 0.015 | 0.200 | 0.024 | 0.144 | 0.067 | **.547^**^** | **.507^**^** | **.362^**^** | **.867^**^** | **.549^**^** | **.496^**^** | **.303^**^** |  |
| **Correlation is significant at the 0.01 level (2-tailed); *Correlation is significant at the 0.05 level (2-tailed). | | | | | | | | | | | | | | | | | | |

| **Table S3. Spearman Correlation Coefficients Between Study Variables of Interest and Covariates** | | | | | | | | | | | | | | | | | | |
| --- | --- | --- | --- | --- | --- | --- | --- | --- | --- | --- | --- | --- | --- | --- | --- | --- | --- | --- |
|  | 1 | 2 | 3 | 4 | 5 | 6 | 7 | 8 | 9 | 10 | 11 | 12 | 13 | 14 | 15 | 16 | 17 | 18 |
| **Covariates** | | | | | | | | | | | | | | | | | | |
| Age | -0.100 | -0.145 | -0.139 | -0.204 | 0.185 | 0.001 | 0.222 | -0.050 | -0.017 | 0.151 | -0.036 | 0.023 | 0.125 | 0.005 | -0.051 | 0.123 | 0.092 | 0.100 |
| Biological Sex | **.330^**^** | 0.227 | -0.120 | -0.161 | 0.222 | 0.145 | **.357^**^** | 0.041 | 0.097 | 0.075 | **.483^**^** | **.408^**^** | **.375^**^** | 0.222 | **.455^**^** | **.383^**^** | **.339^**^** | 0.145 |
| Area Deprivation Index (ADI) National Ranking from 2020 | **.333^**^** | 0.061 | 0.123 | **-.254^*^** | 0.228 | 0.177 | -0.131 | 0.174 | -0.007 | 0.095 | 0.018 | -0.050 | 0.077 | -0.030 | 0.050 | 0.071 | 0.153 | 0.004 |
| Parental Smoking in Home | 0.085 | -0.017 | 0.079 | -0.170 | 0.044 | 0.069 | 0.053 | 0.082 | 0.181 | 0.053 | 0.110 | 0.079 | 0.044 | 0.076 | 0.035 | 0.008 | 0.051 | 0.032 |
| Body Mass Index (BMI) | 0.048 | **.331^**^** | -0.133 | **-.257^*^** | **.659^**^** | 0.015 | **.312^**^** | 0.046 | -0.041 | 0.011 | 0.070 | 0.027 | -0.059 | 0.055 | 0.083 | 0.004 | -0.094 | 0.172 |
| *Please see table S1 for the numbering of variables of interest. **Correlation is significant at the 0.01 level (2-tailed); *Correlation is significant at the 0.05 level (2-tailed).* | | | | | | | | | | | | | | | | | | |

| **Table S4. Spearman Correlation Coefficients Between Covariates** | | | | | |
| --- | --- | --- | --- | --- | --- |
|  | Age | Biological Sex | Area Deprivation Index (ADI) National Ranking from 2020 | Parental Smoking in Home | Body Mass Index (BMI) |
| Age |  | 0.085 | 0.179 | 0.155 | 0.217 |
| Biological Sex | 0.085 |  | **.306^**^** | 0.087 | **.304^**^** |
| Area Deprivation Index (ADI) National Ranking from 2020 | 0.179 | **.306^**^** |  | **.313^**^** | **.451^**^** |
| Parental Smoking in Home | 0.155 | 0.087 | **.313^**^** |  | **.269^*^** |
| Body Mass Index (BMI) | 0.217 | **.304^**^** | **.451^**^** | **.269^*^** |  |
| ***Correlation is significant at the 0.01 level (2-tailed); *Correlation is significant at the 0.05 level (2-tailed).* | | | | | |

| **Table S5. PM_2.5_ and Lipid Mediators** | | | | | | | | | | |
| --- | --- | --- | --- | --- | --- | --- | --- | --- | --- | --- |
|  | PGE2 (ng/mL) | | PGE3 (ng/mL) | | 12(S)-HETE (ng/mL) | | 12(S)-HEPE (ng/mL) | | 15(S)-HETE (ng/mL) | |
| *Predictor* | *β* | *95% CI*  *(Lower, Upper)* | *β* | *95% CI*  *(Lower, Upper)* | *β* | *95% CI*  *(Lower, Upper)* | *β* | *95% CI*  *(Lower, Upper)* | *β* | *95% CI*  *(Lower, Upper)* |
| Past Month PM_2.5_ (µg/m^3^) | 1.20 | **1.07, 1.32** | 1.02 | 0.95, 1.10 | 1.29 | **1.15, 1.48** | 1.23 | **1.12, 1.38** | 1.10 | **1.05, 1.17** |
| Biological Sex | 1.02 | 0.58, 1.82 | 1.55 | **1.07, 2.24** | 0.83 | 0.43, 1.66 | 1.05 | 0.59, 1.91 | 1.00 | 0.72, 1.38 |
| PM_2.5_ by Sex | N.S. | N.S. | N.S. | N.S. | N.S. | N.S. | N.S. | N.S. | N.S. | N.S. |
| Overall Model | *F(6,58)* = 2.78,  ***p* = 0.01** | | *F(6,61)* = 4.79,  ***p* < 0.01** | | *F(6,66)* = 3.53,  ***p* < 0.01** | | *F(6,57)* = 4.08,  ***p* < 0.01** | | *F(6,66)* = 2.95,  ***p* = 0.01** | |
| *Covariates include age, Area Deprivation Index (ADI) National Rank with a >75^th^ percentile cutoff from 2020, Body Mass Index (BMI), and parental smoking in home.  Non-significant interaction terms (denoted as 'N.S.') indicate that the interaction was removed, and the model was rerun with only the main effects.  Confidence intervals are bolded for significant results (p < 0.05).* *Inflammatory markers were log-transformed for analysis. The reported beta coefficients and confidence intervals have been back-transformed to the original scale for ease of interpretation* | | | | | | | | | | |

| **Table S6. PM_2.5_ and Pro-Inflammatory Cytokines and C-Reactive Protein** | | | | | | | | |
| --- | --- | --- | --- | --- | --- | --- | --- | --- |
|  | IL-6 (pg/mL) | | IL-8 (pg/mL) | | TNF-α (pg/mL) | | CRP (mg/L) | |
| *Predictor* | *β* | *95% CI*  *(Lower, Upper)* | *β* | *95% CI*  *(Lower, Upper)* | *β* | *95% CI*  *(Lower, Upper)* | *β* | *95% CI*  *(Lower, Upper)* |
| Past Month PM_2.5_ (µg/m^3^) | 0.89 | **0.79, 0.98** | 1.02 | 0.98, 1.05 | 0.95 | 0.89, 1.01 | 1.09 | 0.98, 1.20 |
| Biological Sex | 0.27 | 0.06, 1.17 | 0.91 | 0.74, 1.10 | 1.04 | 0.74, 1.48 | 1.01 | 0.56, 1.82 |
| PM_2.5_ by Sex | **1.17** | **1.01, 1.35** | N.S. | N.S. | N.S. | N.S. | N.S. | N.S. |
| Overall Model | *F(7,55)* = 3.29,  ***p* = 0.01** | | *F(6,55)* = 0.80,  *p* = 0.58 | | *F(6,55)* = 1.06,  *p* = 0.40 | | *F(6,54)* = 9.16,  ***p* < 0.01** | |
| *Covariates include age, Area Deprivation Index (ADI) National Rank with a >75^th^ percentile cutoff from 2020, Body Mass Index (BMI), and parental smoking in home.  Non-significant interaction terms (denoted as 'N.S.') indicate that the interaction was removed, and the model was rerun with only the main effects.  Confidence intervals are bolded for significant results (p < 0.05). Inflammatory markers were log-transformed for analysis. The reported beta coefficients and confidence intervals have been back-transformed to the original scale for ease of interpretation* | | | | | | | | |

| **Table S7. PM_2.5_ and Psychiatric Symptom Scores** | | | | | | | | |
| --- | --- | --- | --- | --- | --- | --- | --- | --- |
|  | SCARED Total Score | | SCARED Generalized Anxiety Disorder (GAD) Subscore | | SCARED Social Anxiety Disorder Subscore | | CDI Total Score | |
| *Predictors* | *β* | *95% CI*  *(Lower, Upper)* | *β* | *95% CI*  *(Lower, Upper)* | *β* | *95% CI*  *(Lower, Upper)* | *β* | *95% CI*  *(Lower, Upper)* |
| Past Month PM_2.5_ (µg/m^3^) | -0.20 | -1.70, 1.30 | 0.04 | -0.47, 0.54 | -0.05 | -0.46, 0.36 | 1.07 | 0.98, 1.15 |
| PM_2.5_ by Sex | 1.57 | **0.82, 2.31** | 0.44 | **0.19, 0.70** | 0.30 | **0.10, 0.50** | N.S. | N.S. |
| Overall Model | *F(6,66)* = 4.23,  ***p* < 0.01** | | *F(6,66)* = 3.36,  ***p* = 0.01** | | *F(6,66)* = 2.34,  ***p* = 0.04** | | *F(5,67)* = 0.75,  *p* = 0.59 | |
| *Covariates include age, Area Deprivation Index (ADI) National Rank with a >75^th^ percentile cutoff from 2020, Body Mass Index (BMI), and parental smoking in home.  Non-significant interaction terms (denoted as 'N.S.') indicate that the interaction was removed, and the model was rerun with only the main effects. The CDI total score was log-transformed for analysis and thus the reported beta coefficients and confidence intervals have been back-transformed to the original scale for ease of interpretation. The main effect of sex was excluded in the models due to potential collider bias. Confidence intervals are bolded for significant results (p < 0.05). SCARED: Screen for Child Anxiety Related Emotional Disorders; CDI: Children’s Depression Inventory.* | | | | | | | | |

| **Table S8. PGE2 and Psychiatric Symptom Scores** | | | | | | | | |
| --- | --- | --- | --- | --- | --- | --- | --- | --- |
|  | SCARED Total Score | | SCARED Generalized Anxiety Disorder (GAD) Subscore | | SCARED Social Anxiety Disorder Subscore | | CDI Total Score | |
| *Predictors* | *β* | *95% CI*  *(Lower, Upper)* | *β* | *95% CI*  *(Lower, Upper)* | *β* | *95% CI*  *(Lower, Upper)* | *β* | *95% CI*  *(Lower, Upper)* |
| Log_10_(PGE2 (ng/mL)) | 2.72 | -4.60, 10.04 | 1.51 | -1.23, 4.14 | 0.80 | -1.15, 2.75 | 0.91 | 0.58, 1.45 |
| Biological Sex | 16.48 | **9.33, 23.64** | 4.51 | **1.94, 7.07** | 3.55 | **1.64, 5.45** | 1.78 | **1.12, 2.82** |
| PGE2 by Sex | N.S. | N.S. | N.S. | N.S. | N.S. | N.S. | N.S. | N.S. |
| Overall Model | *F(6,58)* = 4.17,  ***p* < 0.01** | | *F(6,58)* = 2.65,  ***p* = 0.02** | | *F(6,58)* = 3.39,  ***p* < 0.01** | | *F(6,58)* = 1.10,  *p* = 0.37 | |
| *Covariates include age, Area Deprivation Index (ADI) National Rank with a >75^th^ percentile cutoff from 2020, Body Mass Index (BMI), and parental smoking in home.  Non-significant interaction terms (denoted as 'N.S.') indicate that the interaction was removed, and the model was rerun with only the main effects. Confidence intervals are bolded for significant results (p < 0.05). The CDI total score was log-transformed for analysis and thus the reported beta coefficients and confidence intervals have been back-transformed to the original scale for ease of interpretation. SCARED: Screen for Child Anxiety Related Emotional Disorders; CDI: Children’s Depression Inventory.* | | | | | | | | |

| **Table S9. PGE3 and Psychiatric Symptom Scores** | | | | | | | | |
| --- | --- | --- | --- | --- | --- | --- | --- | --- |
|  | SCARED Total Score | | SCARED Generalized Anxiety Disorder (GAD) Subscore | | SCARED Social Anxiety  Disorder Subscore | | CDI Total Score | |
| *Predictors* | *β* | *95% CI*  *(Lower, Upper)* | *β* | *95% CI*  *(Lower, Upper)* | *β* | *95% CI*  *(Lower, Upper)* | *β* | *95% CI*  *(Lower, Upper)* |
| Log_10_(PGE3 (ng/mL)) | 0.02 | -12.55, 12.58 | -2.18 | -6.42, 2.05 | 0.31 | -2.97, 3.59 | 1.15 | 0.49, 2.57 |
| Biological Sex | 16.20 | **8.62, 23.78** | 4.89 | **2.34, 7.45** | 3.30 | **1.32, 5.29** | 1.45 | 0.87, 2.34 |
| PGE3 by Sex | N.S. | N.S. | N.S. | N.S. | N.S. | N.S. | N.S. | N.S. |
| Overall Model | *F(6,60)* = 3.87,  ***p* < 0.01** | | *F(6,60)* = 2.94,  ***p* = 0.01** | | *F(6,60)* = 2.66,  ***p* = 0.02** | | *F(6,60)* = 0.68,  *p* = 0.66 | |
| *Covariates include age, Area Deprivation Index (ADI) National Rank with a >75^th^ percentile cutoff from 2020, Body Mass Index (BMI), and parental smoking in home.  Non-significant interaction terms (denoted as 'N.S.') indicate that the interaction was removed, and the model was rerun with only the main effects. Confidence intervals are bolded for significant results (p < 0.05). The CDI total score was log-transformed for analysis and thus the reported beta coefficients and confidence intervals have been back-transformed to the original scale for ease of interpretation. SCARED: Screen for Child Anxiety Related Emotional Disorders; CDI: Children’s Depression Inventory.* | | | | | | | | |

| **Table S10. 12(S)-HETE and Psychiatric Symptom Scores** | | | | | | | | |
| --- | --- | --- | --- | --- | --- | --- | --- | --- |
|  | SCARED Total Score | | SCARED Generalized Anxiety Disorder (GAD) Subscore | | SCARED Social Anxiety  Disorder Subscore | | CDI Total Score | |
| *Predictors* | *β* | *95% CI*  *(Lower, Upper)* | *β* | *95% CI*  *(Lower, Upper)* | *β* | *95% CI*  *(Lower, Upper)* | *β* | *95% CI*  *(Lower, Upper)* |
| Log_10_(12(S)-HETE (ng/mL)) | 2.58 | -2.82, 7.98 | 1.44 | -0.41, 3.28 | 0.76 | -0.70, 2.22 | 1.02 | 0.72, 1.41 |
| Biological Sex | 16.34 | **9.46, 23.23** | 4.39 | **2.03, 6.75** | 3.31 | 1.46, 5.17 | 1.58 | **1.02, 2.45** |
| 12(S)-HETE by Sex | N.S. | N.S. | N.S. | N.S. | N.S. | N.S. | N.S. | N.S. |
| Overall Model | *F(6,65)* = 4.38,  ***p* < 0.01** | | *F(6,65)* = 3.18,  ***p* = 0.01** | | *F(6,65)* = 2.83,  ***p* = 0.02** | | *F(6,65)* = 0.87,  *p* = 0.52 | |
| *Covariates include age, Area Deprivation Index (ADI) National Rank with a >75^th^ percentile cutoff from 2020, Body Mass Index (BMI), and parental smoking in home.  Non-significant interaction terms (denoted as 'N.S.') indicate that the interaction was removed, and the model was rerun with only the main effects. Confidence intervals are bolded for significant results (p < 0.05). The CDI total score was log-transformed for analysis and thus the reported beta coefficients and confidence intervals have been back-transformed to the original scale for ease of interpretation. SCARED: Screen for Child Anxiety Related Emotional Disorders; CDI: Children’s Depression Inventory.* | | | | | | | | |

| **Table S11. 12(S)-HEPE and Psychiatric Symptom Scores** | | | | | | | | |
| --- | --- | --- | --- | --- | --- | --- | --- | --- |
|  | SCARED Total Score | | SCARED Generalized Anxiety Disorder (GAD) Subscore | | SCARED Social Anxiety Disorder Subscore | | CDI Total Score | |
| *Predictors* | *β* | *95% CI*  *(Lower, Upper)* | *β* | *95% CI*  *(Lower, Upper)* | *β* | *95% CI*  *(Lower, Upper)* | *β* | *95% CI*  *(Lower, Upper)* |
| Log_10_(12(S)-HEPE (ng/mL)) | 2.63 | -4.56, 9.82 | 1.65 | -0.82, 4.13 | 0.62 | -1.25, 2.49 | 1.15 | 0.72, 1.82 |
| Biological Sex | 15.41 | **7.85, 22.97** | 3.85 | **1.25, 6.45** | 2.89 | 0.93, 4.86 | 1.38 | 0.85, 2.19 |
| 12(S)-HEPE by Sex | N.S. | N.S. | N.S. | N.S. | N.S. | N.S. | N.S. | N.S. |
| Model Fit | *F(6,56)* = 3.45,  ***p* = 0.01** | | *F(6,56)* = 2.33,  ***p* = 0.04** | | *F(6,56)* = 2.64,  ***p* = 0.03** | | *F(6,56)* = 0.51,  *p* = 0.80 | |
| *Covariates include age, Area Deprivation Index (ADI) National Rank with a >75^th^ percentile cutoff from 2020, Body Mass Index (BMI), and parental smoking in home.  Non-significant interaction terms (denoted as 'N.S.') indicate that the interaction was removed, and the model was rerun with only the main effects. Confidence intervals are bolded for significant results (p < 0.05). The CDI total score was log-transformed for analysis and thus the reported beta coefficients and confidence intervals have been back-transformed to the original scale for ease of interpretation. SCARED: Screen for Child Anxiety Related Emotional Disorders; CDI: Children’s Depression Inventory.* | | | | | | | | |

| **Table S12. 15(S)-HETE and Psychiatric Symptom Scores** | | | | | | | | |
| --- | --- | --- | --- | --- | --- | --- | --- | --- |
|  | SCARED Total Score | | SCARED Generalized Anxiety Disorder (GAD) Subscore | | SCARED Social Anxiety Disorder Subscore | | CDI Total Score | |
| *Predictors* | *β* | *95% CI*  *(Lower, Upper)* | *β* | *95% CI*  *(Lower, Upper)* | *β* | *95% CI*  *(Lower, Upper)* | *β* | *95% CI*  *(Lower, Upper)* |
| Log_10_(15(S)-HETE (ng/mL)) | 5.18 | -6.77, 17.14 | 2.56 | -1.55, 6.68 | 1.15 | -2.08, 4.39 | 1.17 | 0.55, 2.51 |
| Biological Sex | 16.27 | **9.35, 23.18** | 4.37 | **1.99, 6.75** | 3.31 | 1.44, 5.18 | 1.55 | **1.01, 2.40** |
| 15(S)-HETE by Sex | N.S. | N.S. | N.S. | N.S. | N.S. | N.S. | N.S. | N.S. |
| Overall Model | *F(6,65)* = 4.34,  ***p* < 0.01** | | *F(6,65)* = 3.00,  ***p* = 0.01** | | *F(6,65)* = 2.71,  ***p* = 0.02** | | *F(6,65)* = 0.90,  *p* = 0.50 | |
| *Covariates include age, Area Deprivation Index (ADI) National Rank with a >75^th^ percentile cutoff from 2020, Body Mass Index (BMI), and parental smoking in home.  Non-significant interaction terms (denoted as 'N.S.') indicate that the interaction was removed, and the model was rerun with only the main effects. Confidence intervals are bolded for significant results (p < 0.05). The CDI total score was log-transformed for analysis and thus the reported beta coefficients and confidence intervals have been back-transformed to the original scale for ease of interpretation. SCARED: Screen for Child Anxiety Related Emotional Disorders; CDI: Children’s Depression Inventory.* | | | | | | | | |

| **Table S13. IL-6 and Psychiatric Symptom Scores** | | | | | | | | |
| --- | --- | --- | --- | --- | --- | --- | --- | --- |
|  | SCARED Total Score | | SCARED Generalized Anxiety Disorder (GAD) Subscore | | SCARED Social Anxiety Disorder Subscore | | CDI Total Score | |
| *Predictors* | *β* | *95% CI*  *(Lower, Upper)* | *β* | *95% CI*  *(Lower, Upper)* | *β* | *95% CI*  *(Lower, Upper)* | *β* | *95% CI*  *(Lower, Upper)* |
| Log_10_(IL-6 (pg/mL) | 4.37 | -5.85, 14.59 | 2.64 | -1.03, 6.31 | 0.25 | -2.75, 3.26 | 1.48 | 0.74, 2.95 |
| Biological Sex | 5.20 | **10.61, 23.90** | 5.03 | **2.65, 7.42** | 3.56 | **1.61, 5.52** | 1.62 | **1.02, 2.51** |
| IL-6 by Sex | N.S. | N.S. | N.S. | N.S. | N.S. | N.S. | N.S. | N.S. |
| Overall Model | *F(6,56)* = 5.54,  ***p* < 0.01** | | *F(6,56)* = 3.98,  ***p* < 0.01** | | *F(6,56)* = 2.49,  ***p* = 0.03** | | *F(6,56)* = 1.45,  *p* = 0.21 | |
| *Covariates include age, Area Deprivation Index (ADI) National Rank with a >75^th^ percentile cutoff from 2020, Body Mass Index (BMI), and parental smoking in home.  Non-significant interaction terms (denoted as 'N.S.') indicate that the interaction was removed, and the model was rerun with only the main effects. Confidence intervals are bolded for significant results (p < 0.05). The CDI total score was log-transformed for analysis and thus the reported beta coefficients and confidence intervals have been back-transformed to the original scale for ease of interpretation. SCARED: Screen for Child Anxiety Related Emotional Disorders; CDI: Children’s Depression Inventory.* | | | | | | | | |

| **Table S14. IL-8 and Psychiatric Symptom Scores** | | | | | | | | |
| --- | --- | --- | --- | --- | --- | --- | --- | --- |
|  | SCARED Total Score | | SCARED Generalized Anxiety Disorder (GAD) Subscore | | SCARED Social Anxiety Disorder Subscore | | CDI Total Score | |
| *Predictors* | *β* | *95% CI*  *(Lower, Upper)* | *β* | *95% CI*  *(Lower, Upper)* | *β* | *95% CI*  *(Lower, Upper)* | *β* | *95% CI*  *(Lower, Upper)* |
| Log_10_(IL-8 (pg/mL)) | 20.54 | -1.23, 42.32 | 4.76 | -3.33, 12.86 | 5.51 | -0.76, 11.79 | 6.03 | **1.45, 25.12** |
| Biological Sex | 17.96 | **11.40, 24.53** | 5.31 | **2.87, 7.75** | 3.63 | **1.74, 5.52** | 1.70 | **1.10, 2.63** |
| IL-8 by Sex | N.S. | N.S. | N.S. | N.S. | N.S. | N.S. | N.S. | N.S. |
| Overall Model | *F(6,55)* = 6.11,  ***p* < 0.01** | | *F(6,55)* = 3.74,  ***p* < 0.01** | | *F(6,55)* = 3.07,  ***p* = 0.01** | | *F(6,55)* = 2.36,  ***p* = 0.04** | |
| *Covariates include age, Area Deprivation Index (ADI) National Rank with a >75^th^ percentile cutoff from 2020, Body Mass Index (BMI), and parental smoking in home.  Non-significant interaction terms (denoted as 'N.S.') indicate that the interaction was removed, and the model was rerun with only the main effects. Confidence intervals are bolded for significant results (p < 0.05). The CDI total score was log-transformed for analysis and thus the reported beta coefficients and confidence intervals have been back-transformed to the original scale for ease of interpretation. SCARED: Screen for Child Anxiety Related Emotional Disorders; CDI: Children’s Depression Inventory.* | | | | | | | | |

| **Table S15. TNF-α and Psychiatric Symptom Scores** | | | | | | | | |
| --- | --- | --- | --- | --- | --- | --- | --- | --- |
|  | SCARED Total Score | | SCARED Generalized Anxiety Disorder (GAD) Subscore | | SCARED Social Anxiety Disorder Subscore | | CDI Total Score | |
| *Predictors* | *β* | *95% CI*  *(Lower, Upper)* | *Β* | *95% CI*  *(Lower, Upper)* | *β* | *95% CI*  *(Lower, Upper)* | *β* | *95% CI*  *(Lower, Upper)* |
| Log_10_(TNF-α (pg/mL)) | -7.57 | -23.72, 8.58 | -0.26 | -5.76, 4.45 | -3.74 | -8.33, 0.84 | 1.17 | 0.46, 2.97 |
| Biological Sex | 18.72 | **11.98, 25.46** | 4.11 | **2.59, 7.52** | 3.88 | **1.97, 5.79** | 1.58 | **1.01, 2.49** |
| TNF-α by Sex | 30.49 | **2.11, 58.88** | N.S. | N.S. | 9.83 | **1.78, 17.89** | N.S. | N.S. |
| Overall Model | *F(7,53)* = 5.29,  ***p* < 0.01** | | *F(6,54)* = 3.32,  ***p* = 0.01** | | *F(7,53)* = 3.11,  ***p* = 0.01** | | *F(6,54)* = 1.14,  *p* = 0.35 | |
| *Covariates include age, Area Deprivation Index (ADI) National Rank with a >75^th^ percentile cutoff from 2020, Body Mass Index (BMI), and parental smoking in home.  Non-significant interaction terms (denoted as 'N.S.') indicate that the interaction was removed, and the model was rerun with only the main effects. Confidence intervals are bolded for significant results (p < 0.05). The CDI total score was log-transformed for analysis and thus the reported beta coefficients and confidence intervals have been back-transformed to the original scale for ease of interpretation. SCARED: Screen for Child Anxiety Related Emotional Disorders; CDI: Children’s Depression Inventory.* | | | | | | | | |

| **Table S16. CRP and Psychiatric Symptom Scores** | | | | | | | | |
| --- | --- | --- | --- | --- | --- | --- | --- | --- |
|  | SCARED Total Score | | SCARED Generalized Anxiety Disorder (GAD) Subscore | | SCARED Social Anxiety Disorder  Subscore | | CDI Total Score | |
| *Predictors* | *β* | *95% CI*  *(Lower, Upper)* | *β* | *95% CI*  *(Lower, Upper)* | *β* | *95% CI*  *(Lower, Upper)* | *β* | *95% CI*  *(Lower, Upper)* |
| Log_10_(CRP (mg/L)) | 3.59 | -3.80, 10.99 | 2.16 | -0.44, 4.76 | 0.17 | -2.00, 2.35 | 1.10 | 0.66, 1.82 |
| Biological Sex | 16.83 | **9.88, 23.77** | 5.10 | **2.65, 7.54** | 3.63 | 1.59, 5.67 | 1.58 | 0.98, 2.51 |
| CRP by Sex | N.S. | N.S. | N.S. | N.S. | N.S. | N.S. | N.S. | N.S. |
| Overall Model | *F(6,54)* = 5.35,  ***p* < 0.01** | | *F(6,54)* = 4.12,  ***p* < 0.01** | | *F(6,54)* = 2.32,  *p* = 0.05 | | *F(6,54)* = 1.13,  *p* = 0.36 | |
| *Covariates include age, Area Deprivation Index (ADI) National Rank with a >75^th^ percentile cutoff from 2020, Body Mass Index (BMI), and parental smoking in home.  Non-significant interaction terms (denoted as 'N.S.') indicate that the interaction was removed, and the model was rerun with only the main effects. Confidence intervals are bolded for significant results (p < 0.05). The CDI total score was log-transformed for analysis and thus the reported beta coefficients and confidence intervals have been back-transformed to the original scale for ease of interpretation. SCARED: Screen for Child Anxiety Related Emotional Disorders; CDI: Children’s Depression Inventory.* | | | | | | | | |
